# A simple and cost-efficient route to prepare sulfonated dihalo-monomers for synthesizing sulfonated aromatic PEMs†

**DOI:** 10.1039/d4ra06283c

**Published:** 2024-11-21

**Authors:** Nodar Dumbadze, Marco Viviani, Klaus-Dieter Kreuer, Giorgi Titvinidze

**Affiliations:** a Agricultural University of Georgia 240 David Aghmashenebeli Alley Tbilisi 0159 Georgia g.titvinidze@agruni.edu.ge; b Hahn-Schickard Gesellschaft für Angewandte Forschung e.V Georges-Koehler-Allee, 103 79110 Freiburg Im Breisgau Germany; c Max-Planck-Institute for Solid State Research Heisenbergstraße 1 70569 Stuttgart Germany

## Abstract

We present a simple and cost-efficient route for the preparation of sulfonated dihalo-monomers for the synthesis of hydrocarbon ionomers. After conventional monomer sulfonation, excess sulfuric acid is quantitatively removed by neutralization with BaCO_3_. This leads to the precipitation of excess H_2_SO_4_ as insoluble BaSO_4_, which is easily separated from the sulfonated monomers in their soluble Ba-forms by filtration. Compared to conventional methods, the proposed approach leads to higher yields, drastically reduces the number of purification steps, and can easily be expanded to the preparation of other sulfonated monomers. The specific monomers presented here are suitable for the preparation of sulfonated polyarylenes and sulfonated polyphenylenes.

## Abstract 2

A new simple and cost-effective method for the synthesis of sulfonated aromatic monomers is presented, offering a drastic reduction in the number of purification procedures and waste production.

## Graphical abstract

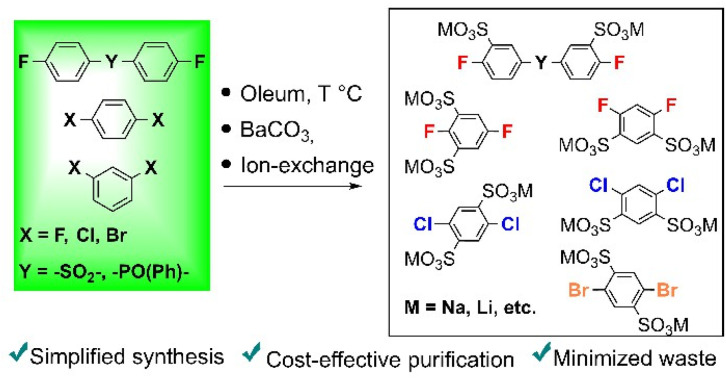

## Introduction

For more than three decades, sulfonated aromatic polymers have been considered alternatives for perfluorosulfonic acid (PFSA) ionomers as membrane materials for fuel cells and electrolyzers. The interest in aromatic membranes was initially driven by cost considerations, but more recent concerns have been related to the use and production of poly- and perfluoro-alkyl substances (PFAS),^1,2^ further increasing the incentive to develop fluorine-free alternatives to benchmark PFSAs.^3^

Initially, progress in this area of research was slow, and this had a lot to do with the unique chemical structure of PFSAs. This combines the extreme hydrophobicity of their PTFE backbones with the hydrophilicity of super-acidic –SO_3_H groups terminating the side chains. These side chains have some flexibility in contrast to the polymer backbone, with their high persistence length stemming from the characteristic helical conformation of PTFE. Over the years, it has become clear that one-to-one aromatic substitutes for PFSAs are surely out of reach: sulfonated aromatic polymers are a separate class of material with distinct properties, offering specific pros and cons compared to PFSAs.^4^ For example, sulfonated hydrocarbons generally have better gas-separating properties and lower electroosmotic water drag, but they can exhibit high swelling in water and lower conductivity at low humidification.

Interestingly, the diverse property profiles of the vast number of aromatic polymer electrolytes developed over the years, with different compositions and architectures, clearly show that specific solutions may meet the requirements of specific well-defined applications.^5–9^ This prospect is closely related to the enormous design-flexibility of sulfonated aromatic polymers, which is in stark contrast to the few degrees of freedom for the design of PFSAs (essentially their ion-exchange capacity and length of the side chains).^7–9^

Two main synthetic strategies enable the synthesis of sulfonated polymers: post-sulfonation of aromatic polymers and polymerization of sulfonated monomers. Historically, sulfonated polymer electrolytes were initially obtained by post-sulfonation. However, the use of sulfonating agents, such as fuming sulfuric acid, concentrated sulfuric acid, or chlorosulfonic acid, was found to cause degradation of the main backbone^10^ and did not allow for the controlled positioning and degree of sulfonation.^8^ To overcome post-sulfonation shortcomings, the synthesis of sulfonated aromatic polymers from presulfonated monomers has become the preferred approach.^11^

Polymerization using sulfonated monomers enables precise control of the sulfonation degree as well as the topological placement along the polymer backbone or side chain.^7^ The sulfonation pattern, *i.e.* the way in which the fixed-ionic groups (–SO_3_H) are distributed within the polymeric structure, has strong effects on the swelling, viscoelastic properties, chemical stability and proton transport. For example, the concentration of sulfonic groups in a domain with high connectivity allows for the combination of high conductivity, low swelling, and reasonable mechanical strength.^12,13^ At the same time, polymer electrolytes obtained from sulfonated monomers have superior stability compared to their postsulfonated analogues, owing to the nature of their sulfonation mechanism.^6,14^ The synthesis of highly sulfonated monomers allows increasing the local density of ionic groups. This may significantly impact the transport properties, especially when used for preparing new multiblock copolymers.^7,13,15^ Accordingly, the conceptualization and development of new sulfonated monomers can play a key role in enabling further progress while paving the way to commercialization.

In this work, we present a new simple, versatile, and adaptable method for the preparation of new and previously reported sulfonated monomers with a high sulfonation degree. Four of these are suitable for the polymerization of sulfonated polyarylenes, such as poly(ether sulfone)s, poly(ether ether ketone)s, poly(phenylene sulfone)s, while three more types of monomer may be used for making sulfonated polyphenylenes.

The presented synthetic route reduces the number of purification steps, and eliminates the need for costly recrystallization and extraction with organic solvents, while minimizing waste production to just a solid barium sulfate cake and a neutral water solution.

## Results and discussion

### Monomers for sulfonated polyarylenes

**Scheme 1:**
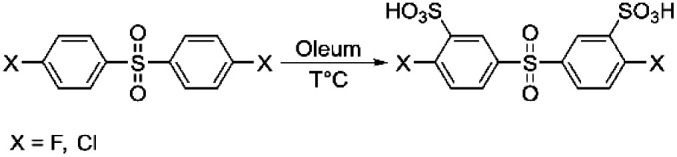
General synthesis scheme for sDCDPS or sDFDPS dihalo-monomers.

Heteroatom-containing sulfonated polyarylenes are generally prepared *via* step-growth polymerization using sulfonated dihalo-monomers. The most commonly used dihalo-monomers are 3,3′-disulfonate-4,4′-dichlorodiphenylsulfone (sDCDPS) and 3,3′-disulfonate-4,4′-difluorodiphenylsulfone (sDFDPS).^16^ Both monomers are prepared *via* electrophilic sulfonation of the corresponding precursors using fuming sulfuric acid (Scheme 1).

Sulfonated dichlorodiphenylsulfone has practical importance because of its relatively low price, while difluoro analogues are more reactive, leading to higher molecular weight polymers with enhanced mechanical properties.^17^ The stronger stabilization of the intermediate Meisenheimer complex is responsible for the higher reactivity of difluoromonomers compared to dichloromonomers.

**Scheme 2:**
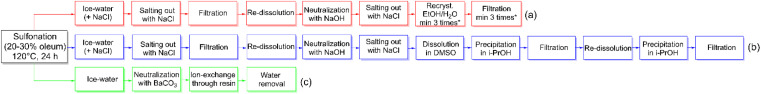
Synthesis of sDFDPS by (a) "conventional" (b) DMSO and (c) BaCO_3_ methods.

The synthesis and purification of sDFDPS are well established.^18–20^ Despite many years of optimization, the procedure involves several steps and at least three recrystallizations,^18^ leading to a minimum of 13 steps (Scheme 2a) and often a questionable purity of the monomer. To reach an acceptable purity for polycondensation reactions, the total yield is usually reduced to around 55 mol%. Here we report an innovative preparation route for sDFDPS and the use of the same method for the synthesis of another six highly sulfonated dihalo-monomers. A new purification process was developed exploiting the solubility of sulfonated monomers in barium form, which we recently reported as a method for the determination of sodium sulfate contamination in sDFDPS.^21^

An alternative to the multiple recrystallizations, the monomer can be separated from the salts using an organic solvent, such as dimethyl sulfoxide (DMSO).^22^ In this case, the insolubility of the salts in DMSO allowed their removal by simple filtration after dissolution of the impure sDFDPS. The monomer obtained by precipitation of the organic solution suffered from DMSO contamination, even after vacuum drying, and needed additional re-precipitation. DMSO contamination is a common problem in this procedure and it can easily be overlooked in ^1^H NMR analysis using DMSO-*d*_6_. However, the contamination was clearly visible in the ^1^H NMR spectrum recorded in D_2_O (ESI Fig. S1†). Although this method effectively removes inorganic salts without the need for recrystallization, it still consists of minimum 12 steps requiring organic solvents (Scheme 2b). The overall yield was increased up to 65%, roughly 10% more than the conventional method.^6^

The new approach developed and proposed here drastically reduces the purification procedure without compromising the purity and yield of the sulfonated monomer. The sulfonation of DFDPS under conventional conditions was followed by direct neutralization with BaCO_3_. The use of carbonate led to a complete removal of excessive H_2_SO_4_, which precipitated as BaSO_4_ and liberated CO_2_, while the filtrate consisted of water-soluble Ba-sDFDPS. After filtration, the monomer was transformed to the desired ion-form by passing the solution through an ion-exchange resin before water removal. The purity of the monomer obtained by this new route (Scheme 2c) was confirmed by ^1^H NMR and it was up to the standard required for polycondensation reactions (ESI Fig. S2–S4†). Possible incomplete exchange from Ba to the Na-form or Li-form was checked by adding Na_2_SO_4_, which did not lead to any precipitate. Overall, this process reduced the work-up times while the total yield was more than 84%. The economic gain of this process is both represented by the saved time and the increased yield.

**Scheme 3:**
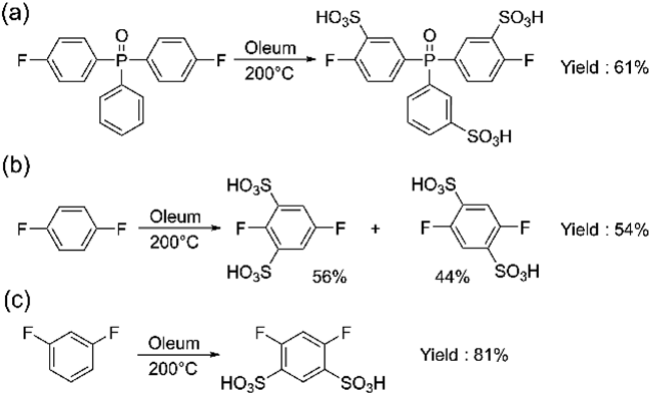
Syntheses of (a) sBFPPO (2), (b) ds-1,4-DFB (3) and (c) ds-1,3-DFB (4).

Another monomer of interest for the synthesis of sulfonated polyarylene is sulfonated bis(4-fluorophenyl)phenyl phosphine oxide (sBFPPO). Due to its high sulfonation degree and higher water solubility, its conventional purification is more complex than for sDFDPS requiring column chromatography. We applied the aforementioned method for synthesizing the sBFPPO monomer (Scheme 3a). The benefit of the proposed method is its versatility for different sulfonated monomers prepared by direct sulfonation and the high yield obtained compared to conventional methods. In the case of sBFPPO, the phosphine oxide instead of sulfone group and the third ring attached to it resulted in a less pure product using the same method. As proven by the ^1^H, ^13^C, and ^19^F NMR spectra (ESI Fig. S5–S7†), an almost pure product was obtained following the same purification procedure. However, small impurities (approx. 2% according to the ^19^F NMR spectrum) were present and this could be assigned to partial sulfonation or sulfonation in different places of the monomer (ESI Fig. S6†). Despite the impurities still being acceptable for condensation polymerization, their presence underlines the impact of the starting monomer on the quality of the resulting product and the need for optimization of the sulfonation step. sBFPPO was obtained in a higher yield of 61.3% compared to previous reports of 50% obtained after column chromatography.^23^

Aiming to expand the monomer portfolio available for the synthesis of sulfonated polyarylenes, we applied the newly developed method to obtain disulfonated difluorophenylenes as potential new building blocks for polymers and block copolymers. A high local sulfonation degree is beneficial for achieving high proton conductivity and nanophase separation, although a trade-off with water solubility is needed for the final polymers. Since “hypersulfonated” units are not beneficial due to counter-ion condensation,^24^ in this work, we aimed for disulfonated monomers without sulfonic groups in the *ortho* position (Schemes 3b and c).

To achieve double sulfonation on the same ring, higher temperatures and longer sulfonation times were required compared to DFDPS or DCDPS (the commonly used temperature range of 110–140 °C for monomers containing a single benzene ring led to a mixture of mono- and disulfonated products, which will not be discussed in detail). The same synthetic steps reported in Scheme 3a were followed. The obtained products and their relative compositions depended on the starting non-sulfonated monomer. In the case of *p*-difluorobenzene, a mixture with a molar ratio of 0.56 : 0.44 of 2,5-difluorobenzene-1,3-disulfonate and 2,5-difluorobenzene-1,4-disulfonate was obtained (Scheme 3b) (see ESI Fig. S8†). In order to separate the isomers, we carried out recrystallization from an i-PrOH/H_2_O mixture, which led to the pure 2,5-difluorobenzene-1,3-disulfonate (54%) (3), while we were not able to separate 2,5-difluorobenzene-1,4-disulfonate in a pure form. On the other hand, *m*-difluorobenzene yielded only 4,6-difluorobenzene-1,3-disulfonate (4) (Scheme 3c) with a high yield of 81%.

The purity of the monomers was confirmed by their ^1^H, ^13^C, and ^19^F NMR spectra (ESI Fig. S9–S14†). The single signal in ^19^F NMR spectrum of 4,6-difluorobenzene-1,3-disulfonate (4) indicated a symmetric substitution (ESI Fig. S13†), while the ^19^F NMR spectrum of 2,5-difluorobenzene-1,3-disulfonate (3) displayed two signals (ESI Fig. S10†). Similar to sDFDPS, full exchange from Ba to the Na- or Li-form was checked by adding Na_2_SO_4_, leading to no precipitation.

### Monomers for sulfonated polyphenylenes

**Scheme 4:**
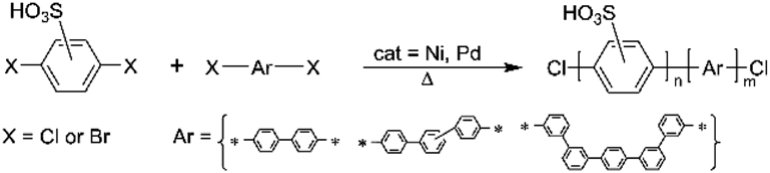
General scheme for the synthesis of sulfonated poly(phenylenes).

The synthesis of sulfonated polyphenylenes (sPP) often involves monomers, such as sulfonated dibromo or dichloro monomers (Scheme 4).^25–33^ These polymers are generally prepared *via* metal-catalyzed reactions, including Ullmann coupling or Ni(0)-mediated coupling.^31,34,35^ Among them, the sulfonated polyphenylenes prepared using 2,5-dichlorobenzene sulfonic acid by Miyatake's group at the University of Yamanashi are the most promising.^29,36^

However, there are a few reports using disulfonated dichloro- or dibromo-benzenes.^32,33,37–42^ In contrast to 2,5-dichlorobenzene sulfonic acid, the disulfonated analogue is not commercially available, which could be due to the complex purification procedure. The high-water solubility of the monomer hinders the efficient salting-out purification step after neutralization, and, for this reason, sometimes this step is simply skipped. However, to remove inorganic salts, extraction with DMF is common practice,^38^ even if sodium sulfate and sodium carbonate are somewhat soluble in DMF,^43^ thereby requiring subsequent Soxhlet extraction with ethanol.^38^

In this work, we implemented the new synthetic approach also to single-ring dihalobenzene compounds to provide a solution to the aforementioned shortcomings.

**Scheme 5:**
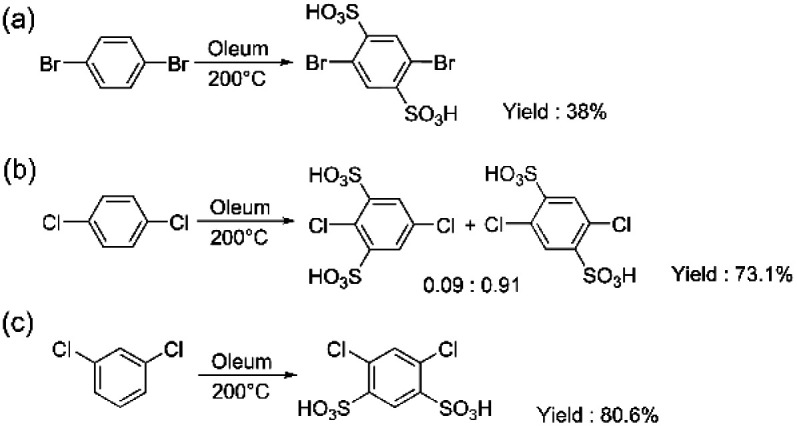
Sulfonation of (a) 1,4-dibromobenzene (5), (b) 1,4-dichlorobenzene (6) and (c) 1,3-dichlorobenzene (7).

After the sulfonation of *p*-dibromobenzene, the excess sulfonic acid was removed by neutralization with BaCO_3_. The water solution containing the sulfonated monomer was passed through an ion-exchange resin to obtain the desired ion-form, in this case the Na-form. The crude product consisted of a mixture of 2,5-dibromobenzene-1,3-disulfonate, 2,5-dibromobenzene-1,4-disulfonate, and a minor amount of degradation products. Due to the overlap of the main signals in the ^1^H NMR spectrum (ESI Fig. S15†), it was impossible to identify the isomers, while the ^13^C NMR spectrum clearly showed the presence of two isomers (ESI Fig. S16†). To separate the isomers, we carried out recrystallization from ethanol/water. This yielded pure 2,5-dibromobenzene-1,4-disulfonate (Scheme 5a) (5) (38%), but separation of pure 2,5-dibromobenzene-1,3-disulfonate was not possible. The purity of the monomer was confirmed by its ^1^H and ^13^C NMR spectra (ESI Fig. S17 and S18†).

Similar to difluoro and dibromo analogues, high temperature and a relatively long sulfonation time were applied for the sulfonation of *p*- and *m*-dichlorobenzenes (Scheme 5b and c). The purification of both monomers was carried out using the aforementioned method. Here, *p*-dichlorobenzene yielded a mixture of 1,4-sulfonated and 1,3 sulfonated products (6) with a ratio of 0.91 : 0.09 and a yield of 73.1%. Further separation of the mixture was not carried out. For *m*-dichlorobenzene, however, the yield of the pure 1,3-sulfonated product (7) was higher (80.6%), similar to the difluoro analogue. For both products, no further purification was needed. The purity of the monomers was confirmed by their ^1^H and ^13^C NMR spectra (ESI Fig. S19–S22†).

In the IR spectra (ESI Fig S23–S29†) of all the synthesized monomers, a new characteristic band at 1025–1041 cm^−1^, assigned to the O=S=O stretching, appeared, indicating successful sulfonation, along with the previously discussed NMR spectral data.

## Experimental section

### Materials

4,4′-Difluorodiphenylsulfone (DFDPS) was supplied by Fumatech BWT GmbH (Germany). 1,3-Difluorobenzene (99%, Alfa Aesar), 1,4-difluorobenzene (99+%, Thermo Scientific), 1,4-dibromobenzene (99%, Thermo Scientific), 1,3-dichlorobenzene (98%, Thermo Fischer), 1,4-dichlorobenzene (99+%, Thermo Scientific), and sulfuric acid fuming (20–30%, Thermo Scientific) were used without further purification. Bis(4-fluorophenyl)phenyl phosphine oxide (BFPPO) was synthesized according to the reported procedure.^44^

### NMR spectroscopy

^1^H, ^13^C, and ^19^F NMR spectra were recorded using Bruker 250 MHz NMR and Bruker 300 MHz NMR spectrometers at room temperature with D_2_O as a solvent and internal standard.

### FT-IR analysis

Infrared spectra were recorded on a Bruker Alpha II FT-IR spectrophotometer with ZnSe-ATR.

### Disodium 3,3′-disulfonate-4,4′-difluorodiphenylsulfone (sDFDPS) (1)

A 50 ml round-bottom flask equipped with a magnetic stirring bar and condenser was charged with DFDPS (5.04 g, 19.8 mmol) and fuming sulfuric acid (11 ml, 20–30% SO_3_). The mixture was heated for 24 h at 140 °C. To avoid a loss of SO_3_, the system was connected to a gas-bubbler filled with silicon oil. After 24 h, the reaction mixture was cooled down and slowly poured into 100 ml of ice water. To the solution, BaCO_3_ was added to precipitate excess sulfuric acid. The solution containing barium 3,3′-disulfonate-4,4′-difluorodiphenylsulfone was concentrated using a rotary evaporator and then passed through an ion-exchange resin (Na-form) leading to disodium 3,3′-disulfonate-4,4′-difluorodiphenylsulfone. Water was distilled off, and the white-coloured monomer was dried in a vacuum oven at 145 °C. The yield was 7.64 g (84%).

^1^H NMR (300 MHz, D_2_O) *δ* 8.41 (dd, *J* = 6.3, 2.4 Hz, 1H), 8.19 (ddd, *J* = 8.8, 4.4, 2.5 Hz, 1H), 7.53 (dd, *J* = 9.6, 8.8 Hz, 1H). ^13^C NMR (75 MHz, D_2_O) *δ* 162.01 (d, *J* = 261.2 Hz), 135.53 (d, *J* = 3.6 Hz), 133.68 (d, *J* = 10.8 Hz), 131.67 (d, *J* = 17.1 Hz), 128.67 (d, *J* = 3.2 Hz), 118.99 (d, *J* = 23.9 Hz). ^19^F NMR (282 MHz, D_2_O) *δ* −102.55 (ddd, *J* = 9.6, 6.4, 4.5 Hz).

### Sulfonated bis(4-fluorophenyl)phenyl phosphine oxide (sBFPPO) (2)

sBFPPO was prepared following the same procedure described above. The yield was 61.3%.

^1^H NMR (300 MHz, D_2_O) *δ* 8.23–8.04 (m, 2H), 7.98–7.72 (m, 2H), 7.55 (ddd, *J* = 10.5, 8.5, 2.2 Hz, 1H). ^13^C NMR (75 MHz, D_2_O) *δ* 161.88 (dd, *J* = 259.9, 3.3 Hz), 143.65 (d, *J* = 12.9 Hz), 138.01 (dd, *J* = 12.2, 10.2 Hz), 134.77 (d, *J* = 11.1 Hz), 131.27 (dd, *J* = 16.0, 13.6 Hz), 130.52, 130.25 (d, *J* = 12.6 Hz), 129.82 (d, *J* = 110.2 Hz), 128.65 (d, *J* = 11.6 Hz), 125.33 (dd, *J* = 109.9, 3.9 Hz), 118.45 (dd, *J* = 22.7, 13.6 Hz). ^19^F NMR (282 MHz, D_2_O) *δ* −104.13 (dt, *J* = 10.6, 5.5 Hz).

### 2,5-Difluorobenzene-1,3-disulfonate (ds-1,4-DFB) (3)

A 50 ml round-bottom flask equipped with a magnetic stirring bar and condenser was charged with 1,4-difluorobenzene (1,4-DFB) (5.00 g, 43.9 mmol) and fuming sulfuric acid (20 ml, 20–30% SO_3_). The mixture was slowly heated to 80 °C and kept for 1 h at this temperature to prevent the evaporation of 1,4-DFB, and then it was heated for 48 h at 200 °C. The further work-up was done as described for sDFDPS. After ion-exchange, the mixture consisted of two isomers: 2,5-difluorobenzene-1,3-disulfonate and 2,5-difluorobenzene-1,4-disulfonate with a ratio of 0.56 : 0.44. To separate the isomers, we carried out recrystallization from an i-PrOH/H_2_O mixture (8 : 1), which yielded pure 2,5-difluorobenzene-1,3-disulfonate with a 54% yield (white powder).

^1^H NMR (300 MHz, D_2_O) *δ* 7.80 (dd, *J* = 7.6, 5.0 Hz, 1H). ^13^C NMR (75 MHz, deuterium oxide) *δ* 156.67 (dd, *J* = 247.8, 3.1 Hz), 151.33 (dd, *J* = 252.9, 3.0 Hz), 132.85 (dd, *J* = 18.7, 6.5 Hz), 118.70 (dd, *J* = 26.7, 2.0 Hz). ^19^F NMR (282 MHz, deuterium oxide) *δ* −115.17 (dt, *J* = 18.8, 7.6 Hz), −118.11 (dt, *J* = 18.9, 5.1 Hz).

### 4,6-Difluorobenzene-1,3-disulfonate (ds-1,3-DFB) (4)

ds-1,3-DFB was prepared following the procedure described above, but with a shorter reaction time (15 h). In contrast to above, a 50 ml round-bottom flask equipped with a magnetic stirring bar and condenser was charged with 1,3-difluorobenzene (1,3-DFB) (5.01 g, 43.9 mmol) and fuming sulfuric acid (20 ml, 20–30% SO_3_). The mixture was slowly heated to 80 °C and kept for 1 h at this temperature to prevent the evaporation of 1,3-DFB, and then it was heated for 15 h at 200 °C. The further work-up was done as described for sDFDPS. The yield was 11.31 g (81%) white powder.

^1^H NMR (300 MHz, D_2_O) *δ* 8.29 (t, *J* = 7.8 Hz, 1H), 7.37 (t, *J* = 9.8 Hz, 1H). ^13^C NMR (75 MHz, D_2_O) *δ* 160.90 (dd, *J* = 259.5, 13.6 Hz), 128.91, 126.54 (dd, *J* = 11.8, 8.4 Hz), 107.08 (t, *J* = 26.8 Hz). ^19^F NMR (282 MHz, D_2_O) *δ* −102.40 (dd, *J* = 9.8, 7.9 Hz).

### 2,5-Dibromobenzene-1,4-disulfonate (ds-1,4-DBB) (5)

ds-1,4-DBB was prepared following the procedure described above, but with a reaction time of 24 h. After ion-exchange, the mixture consisted of two isomers: 2,5-dibromobenzene-1,3-disulfonate and 2,5-dibromobenzene-1,4-disulfonate. To separate the isomers, we carried out recrystallization from an EtOH/H_2_O mixture (7 : 1), which yielded pure 2,5-dibromobenzene-1,4-disulfonate with a 38% yield.

^1^H NMR (300 MHz, D_2_O) *δ* 8.39 (s). ^13^C NMR (75 MHz, D_2_O) *δ* 144.77, 135.06, 118.14.

### 2,5-Dichlorobenzene-1,4-disulfonate (1,4-ds-2,5-DCB)/2,5-dichlorobenzene-1,3-disulfonate (1,3-ds-2,5-DCB) (6)

The sulfonation of 1,4-dichlorobenzene was carried out following the procedure described above. After ion-exchange, the obtained mixture consisted of 1,4-sulfonated and 1,3-sulfonated products with a ratio of 0.91 : 0.09. No further purification was done. The yield was 73.1%.

^1^H NMR (300 MHz, D_2_O) *δ* 8.24 (s) (1,4-ds-2,5-DCB), *δ* 8.20 (s) (1,3-ds-2,5-DCB). ^13^C NMR (75 MHz, D_2_O) *δ* 143.28, 132.64, 131.61 (1,4-ds-2,5-DCB) and 143.00, 131.71, 129.62, 127.03 (1,3-ds-2,5-DCB).

### 4,6-Dichlorobenzene-1,3-disulfonate (ds-1,3-DCB) (7)

ds-1,3-DCB was prepared following the procedure described above. After ion-exchange, no further purification was needed. The yield was 80.6%.

^1^H NMR (250 MHz, D_2_O) *δ* 8.61 (s, 1H), 7.95 (s, 1H). ^13^C NMR (75 MHz, D_2_O) *δ* 138.64, 134.50, 133.89, 129.15.

## Conclusions

The newly developed method for the synthesis of mono-, di-, and tri-sulfonated monomers offers a simpler, more economical, and sustainable purification route after a common sulfonation procedure. The new method offers three main advantages compared to conventional purification systems: (i) simplicity, since no recrystallization is needed, no extractions and no organic solvents are employed; (ii) use of cheap and easily available chemicals with a reduction of waste, which is simply limited to a solid cake and a neutral water solution; and (iii) drastic reduction of the number of purification steps from 13 to 6, which comes with a significant reduction of the time and costs. Overall, the proposed monomer synthesis appears to be versatile and adaptable to different sulfonated monomers independent of the degree of sulfonation and the presence of heteroatoms. This synthetic strategy will enable a faster and simpler way to expand the portfolio of sulfonated monomers for the preparation of sulfonated aromatic polymers and block copolymers. As substitutes for PFSAs, they will play a key role in the future of the emerging hydrogen economy.

## Data availability

The data supporting this article have been included as part of the ESI.† This includes ^1^H, ^13^C and ^19^F NMR and IR spectral data.

## Conflicts of interest

There are no conflicts to declare.

## Supplementary Material

RA-014-D4RA06283C-s001
