# Supplementary material for: A simple and cost-efficient route to prepare sulfonated dihalo-monomers for synthesizing sulfonated aromatic PEMs
Source: RSC Adv. 2024 Nov 21;14(50):37272–7. doi: 10.1039/d4ra06283c (PMC11580002; doi:10.1039/d4ra06283c)
Supplement: RA-014-D4RA06283C-s001 [file RA-014-D4RA06283C-s001.pdf]

Supplementary Information (SI) for RSC Advances.
This journal is © The Royal Society of Chemistry 2024

Supplementary Information

A simple and cost-efficient route to sulfonated dihalo monomers: building blocks for sulfonated aromatic PEMs

Nodar Dumbadze,[a] Marco Viviani,[b] Klaus-Dieter Kreuer[b,c] and Giorgi Titvinidze*[a,b]

[a] Agricultural University of Georgia, 240 David Aghmashenebeli Alley, Tbilisi 0159, Georgia.
g.titvinidze@agruni.edu.ge

[b] Hahn-Schickard Gesellschaft für Angewandte Forschung e.V, Georges-Koehler-Allee, 103, 79110, Freiburg im Breisgau, Germany.

[c] Max-Planck-Institute for Solid State Research, Heisenbergstraße 1, 70569 Stuttgart, Germany.

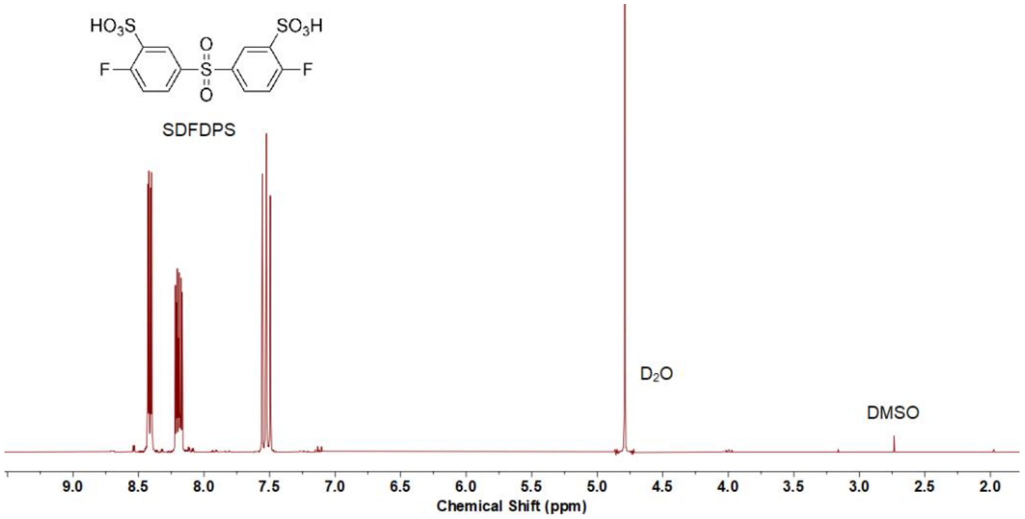

Fig. S 1 ^{1}H-NMR in D_2O showing DMSO contamination of sDFDPS

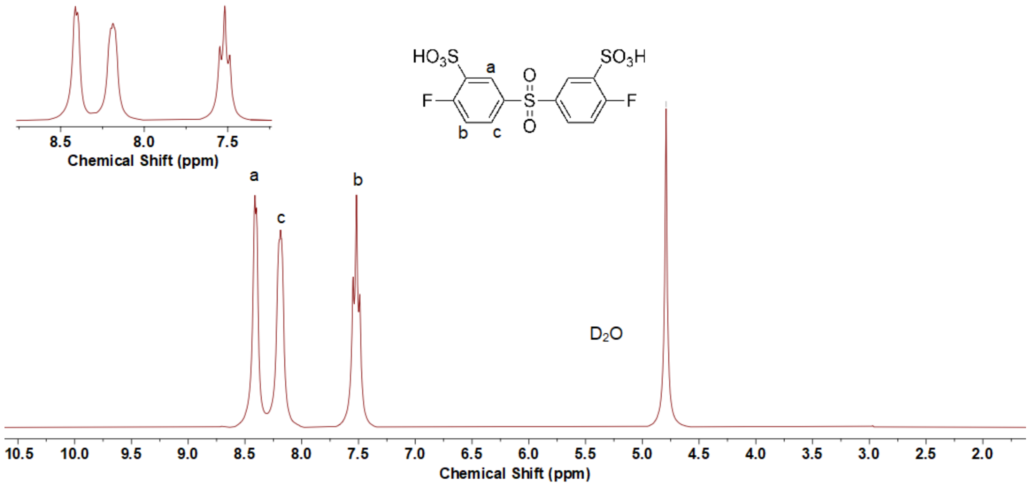

Fig. S 2 ^{1}H-NMR in D_2O of pure sDFDPS (1) showing absence of DMSO and related proton multiplets

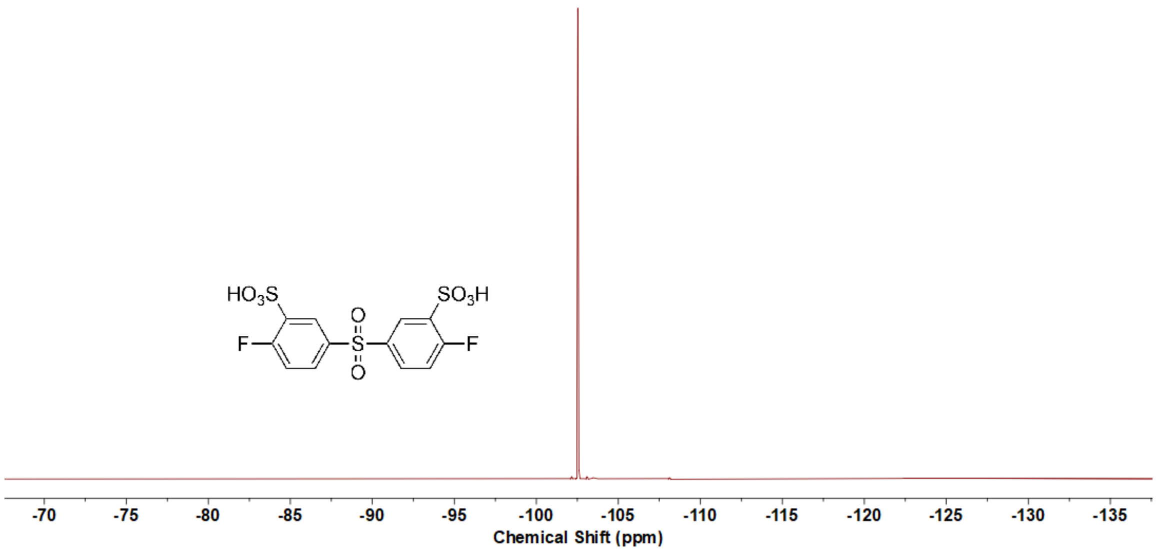

Fig. S 3 ^{19}F NMR in D_2O of pure sDFDPS (1)

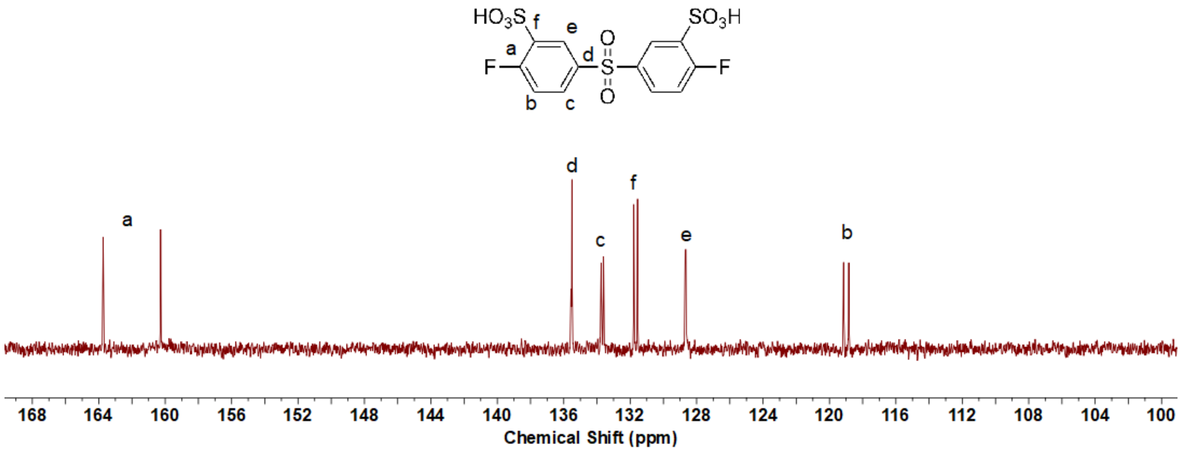

Fig. S 4 ^{13}C-NMR in D_2O of pure sDFDPS (1)

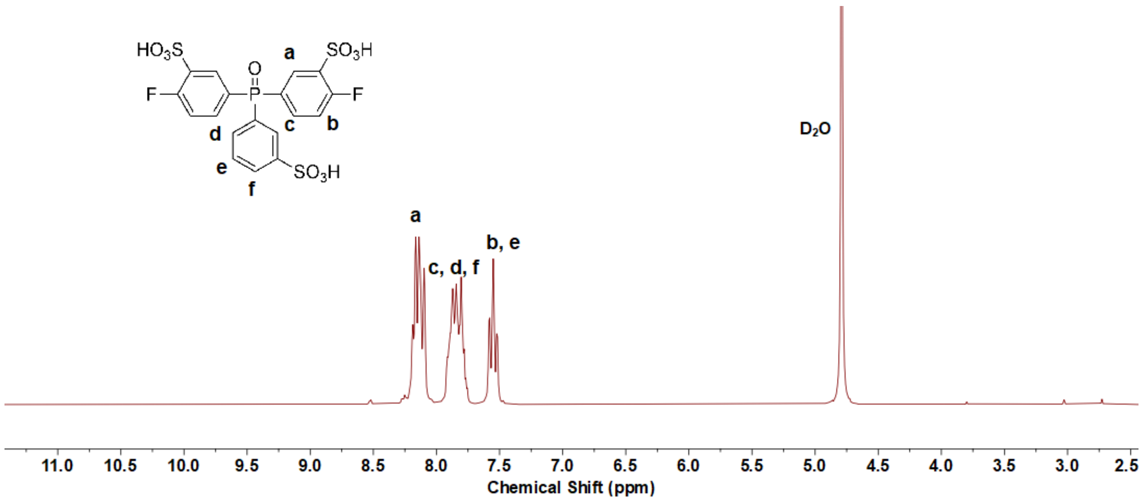

Fig. S 5 ^{1}H-NMR of sBFPPO (2) in D_2O

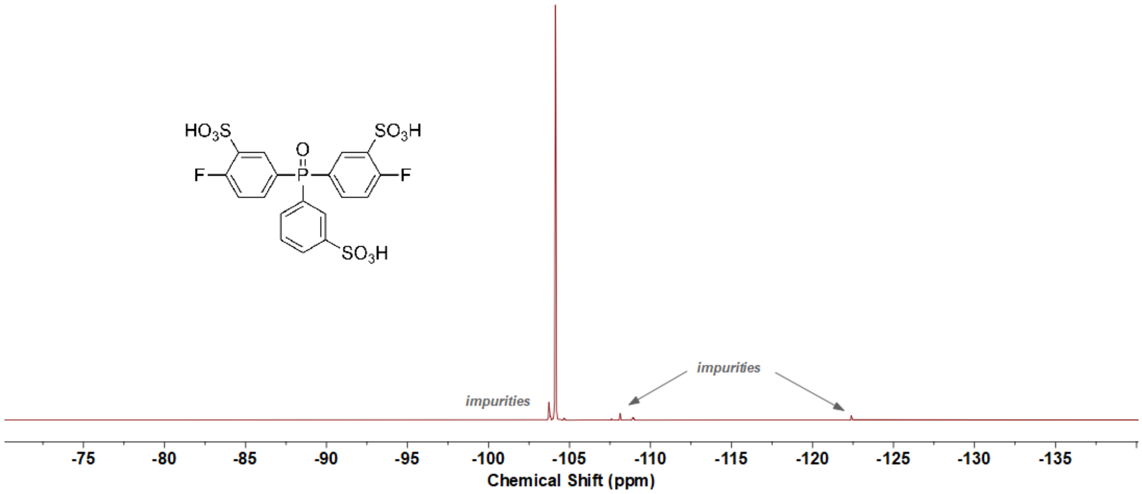

Fig. S 6 ^{19}F NMR of sBFPPO (2) in D_2O showing the presence of traces impurities (ca. 2% mol)

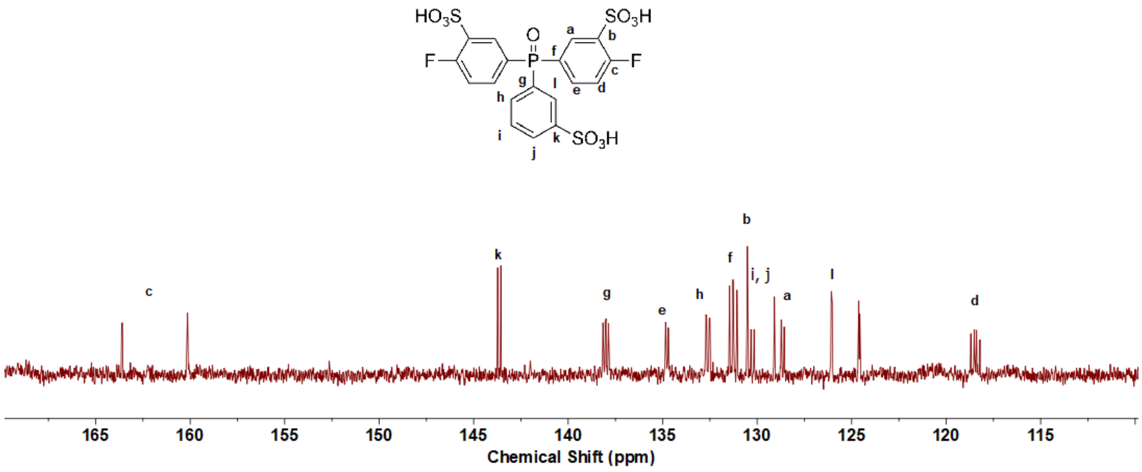

Fig. S 7 ^{13}C NMR of sBFPPO (2) in D_2O

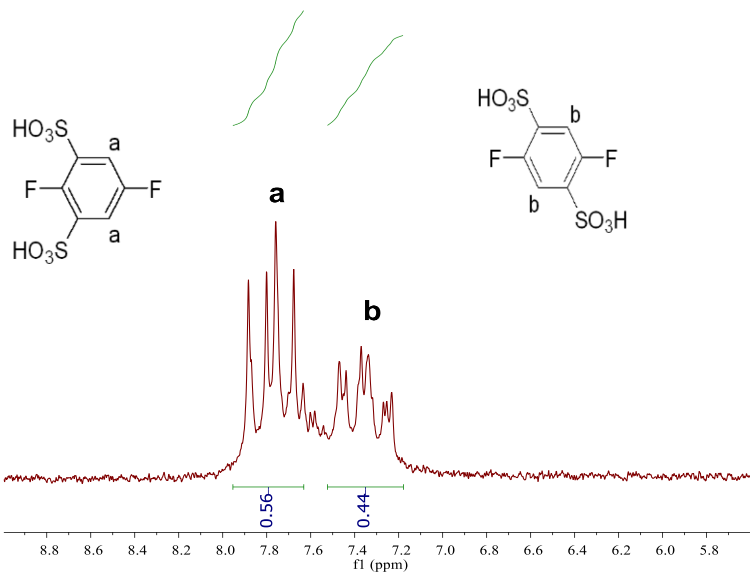

Fig. S 8 ^{1}H NMR in D_2O of crude mixture of 2,5-difluorobenzene-1,3-disulfonate and 2,5-difluorobenzene-1,4-disulfonate obtained after sulfonation

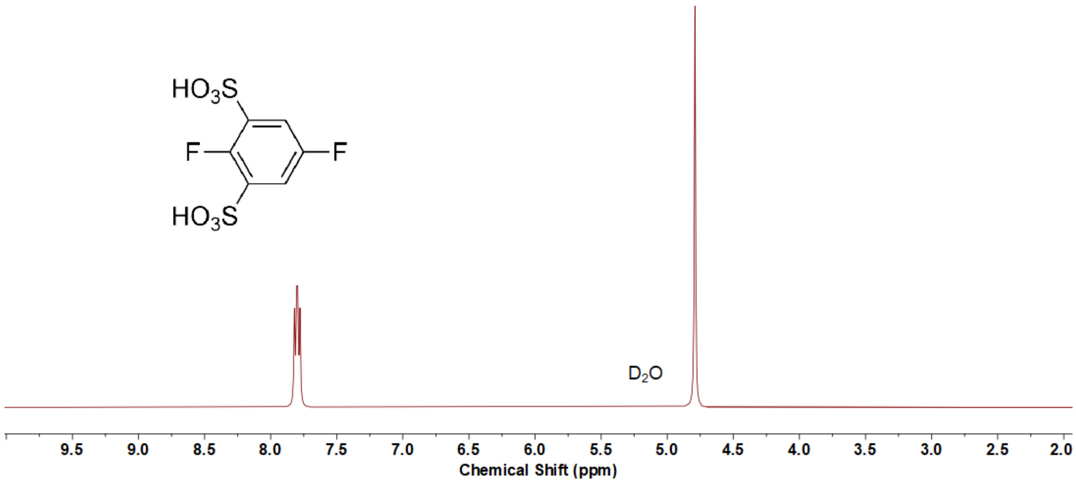

Fig. S 9 ^{1}H NMR in D_2O of pure 2,5-difluoro-1,3-benzenedisulfonic acid (3)

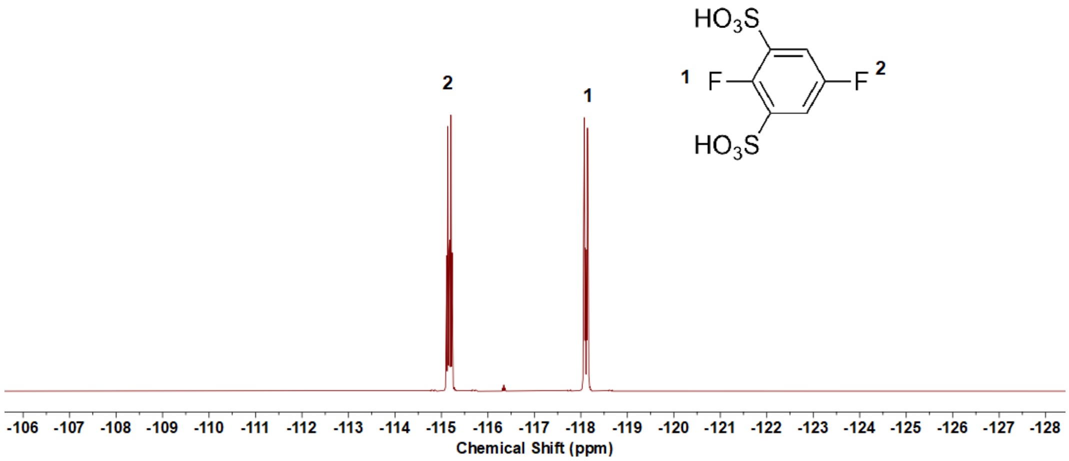

Fig. S 10 ^{19}F NMR in D_2O of pure 2,5-difluoro-1,3-benzenedisulfonic acid (3)

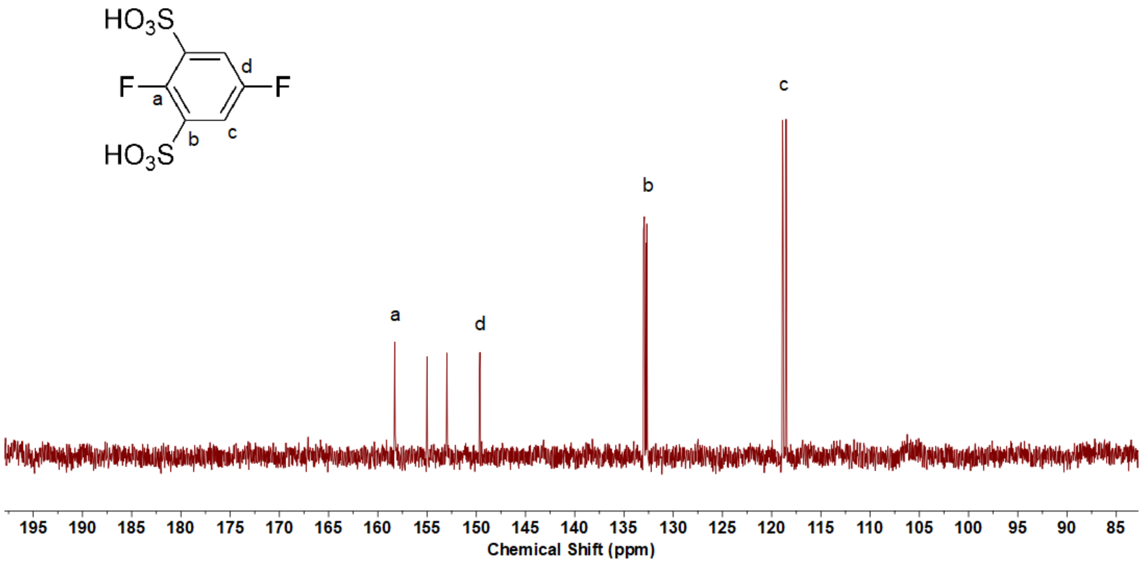

Fig. S 11 ^{13}C NMR in D_2O of pure 2,5-difluoro-1,3-benzenedisulfonic acid (3)

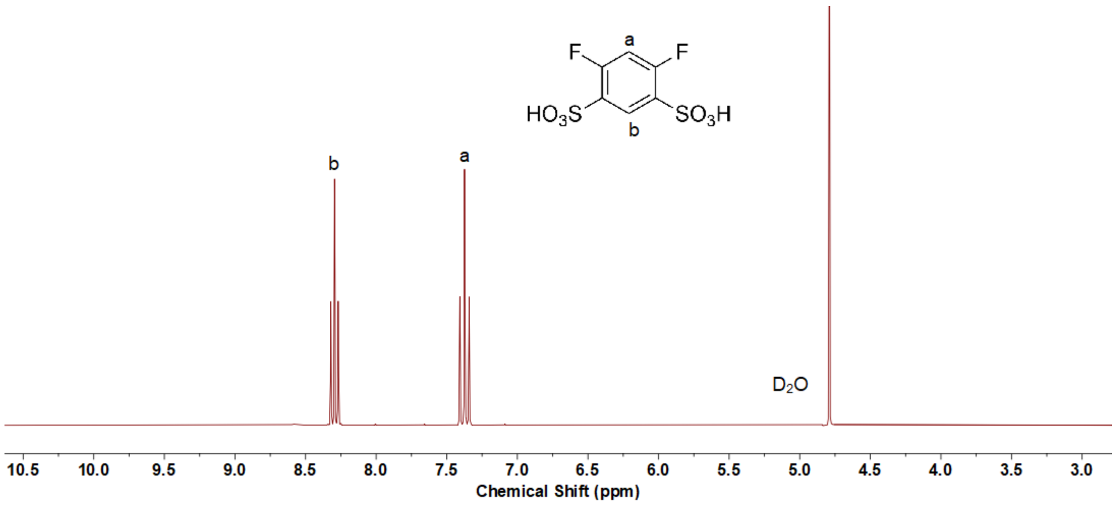

Fig. S 12 ^{1}H NMR in D_2O of pure 4,6-difluoro-1,3-benzenedisulfonic acid (4)

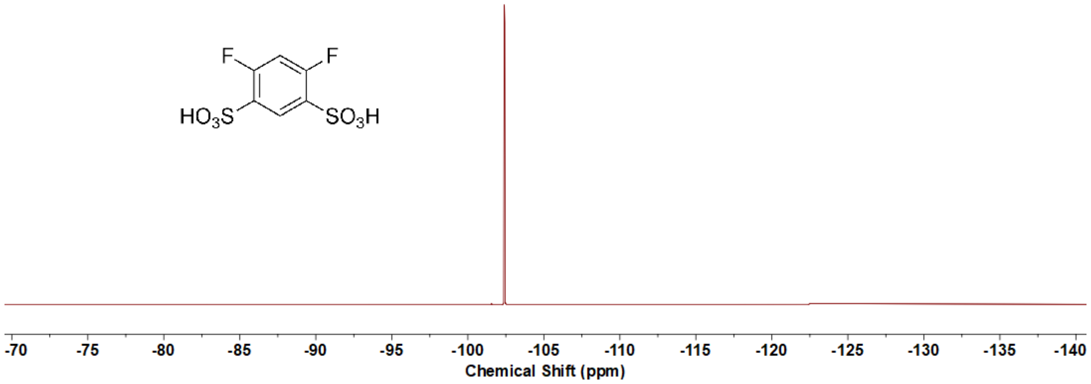

Fig. S 13 ^{19}F NMR in D_2O of pure 4,6-difluoro-1,3-benzenedisulfonic acid (4)

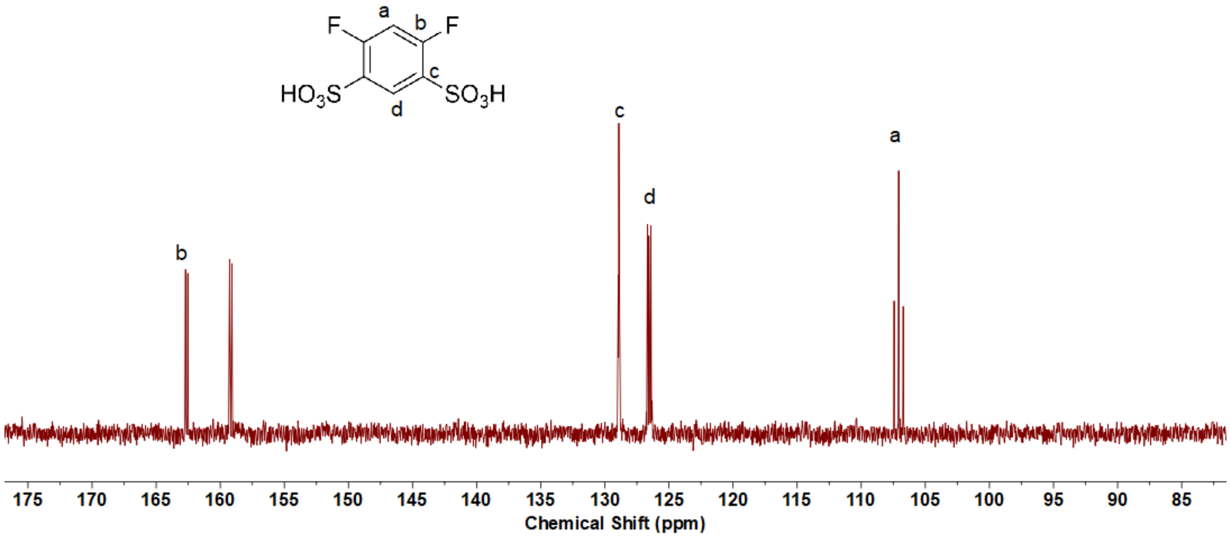

Fig. S 14 ^{13}C NMR in D_2O of pure 4,6-difluoro-1,3-benzenedisulfonic acid (4)

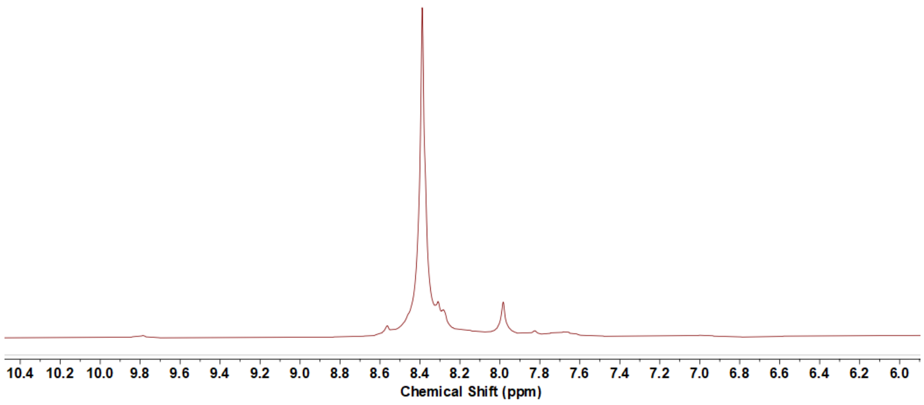

Fig. S 15 ^{1}H NMR in D_2O of crude product of 1,4-dibromobenzene sulfonation before purification

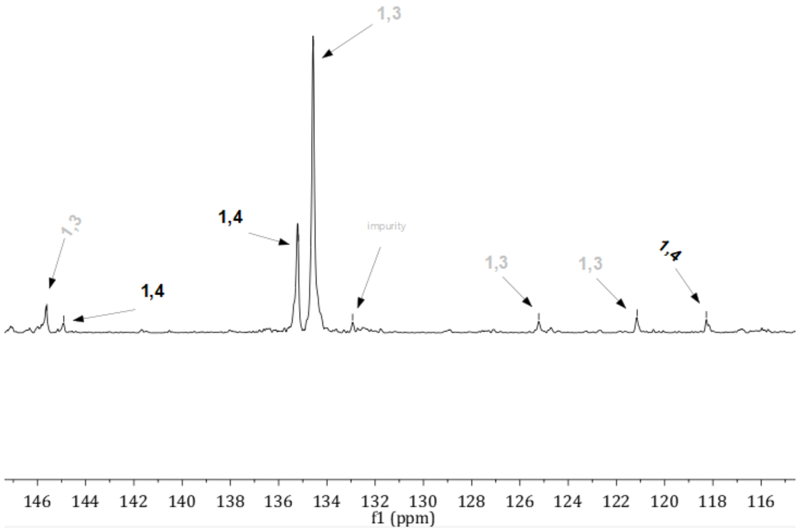

Fig. S 16 ^{13}C NMR in D_2O of crude product of 1,4-dibromobenzene sulfonation before purification

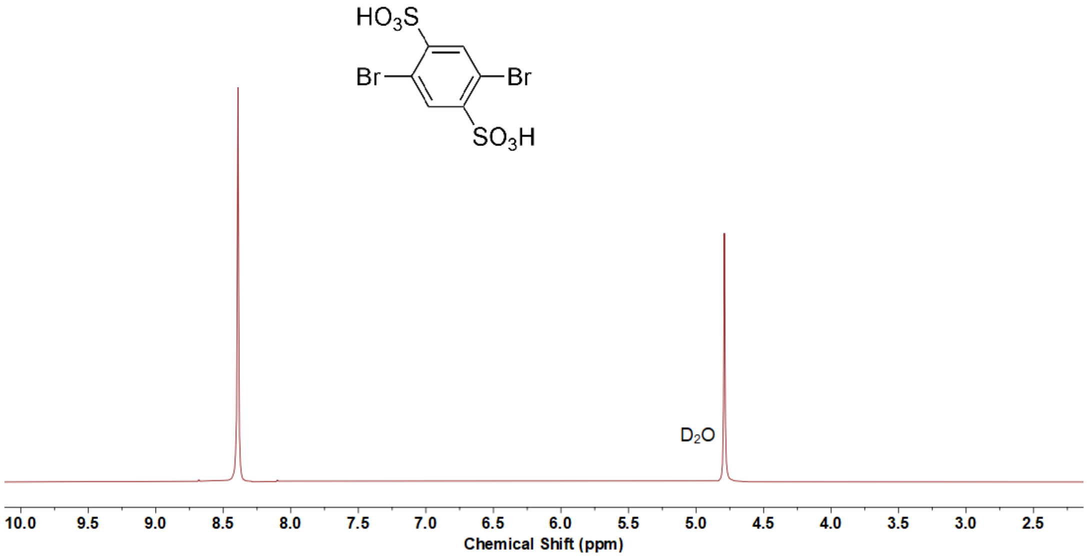

Fig. S 17 ^{1}H NMR in D_2O of pure 2,5-dibromo-1,4-benzenedisulfonic acid (5)

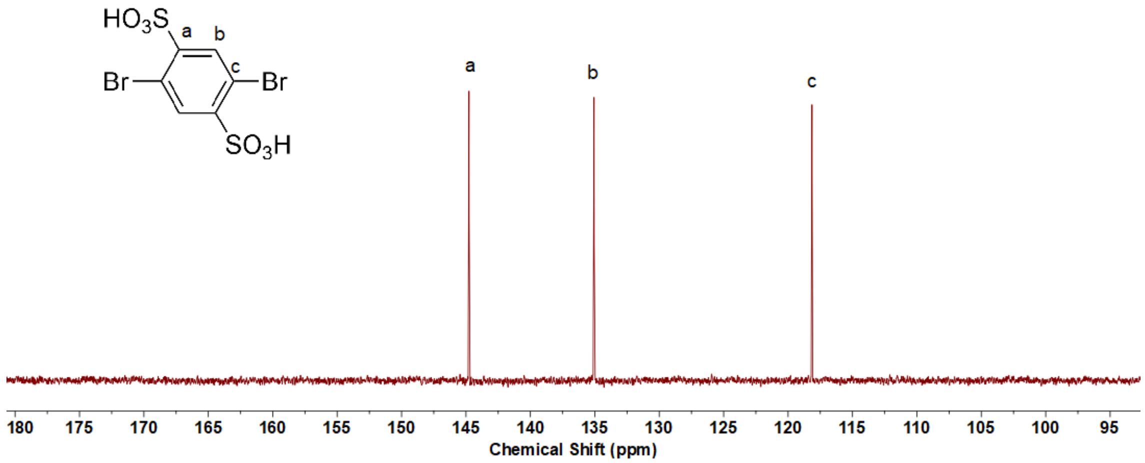

Fig. S 18 ^{13}C NMR in D_2O of pure 2,5-dibromo-1,4-benzenedisulfonic acid (5)

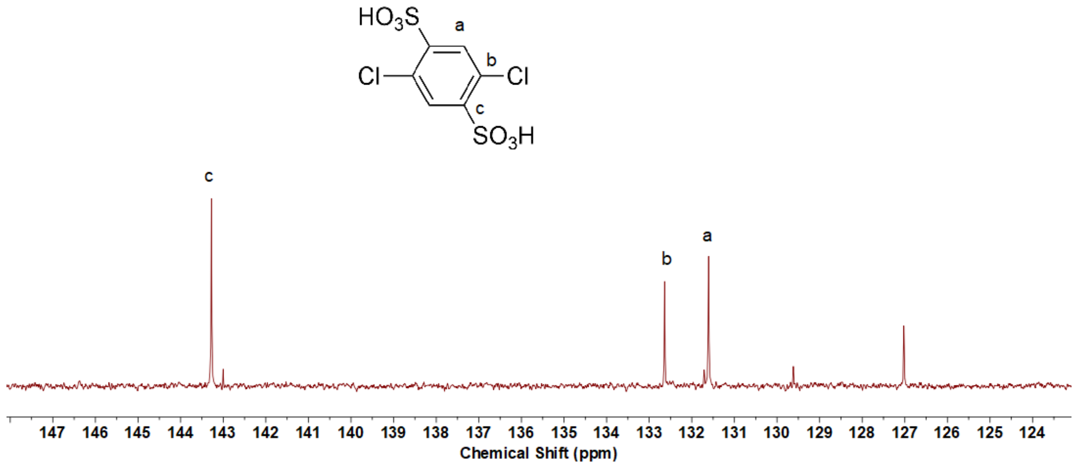

Fig. S 19 ^{13}C NMR in D_2O of the mixture of 2,5-dichlorobenzene-1,4-disulfonate (1,4-ds-2,5-DCB) and 2,5-dichlorobenzene-1,3-disulfonate (1,3-ds-2,5-DCB) (6)

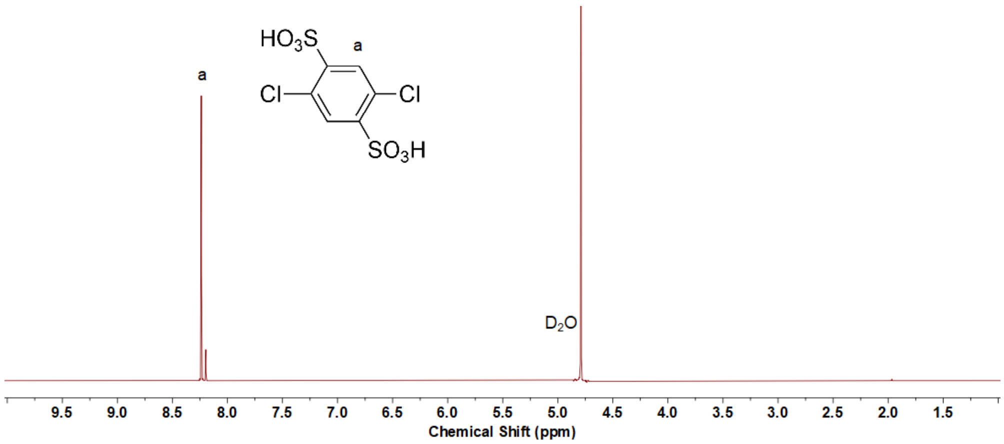

Fig. S 20 ^{1}H NMR of the mixture of 2,5-dichlorobenzene-1,4-disulfonate (1,4-ds-2,5-DCB) and 2,5-dichlorobenzene-1,3-disulfonate (1,3-ds-2,5-DCB) (6)

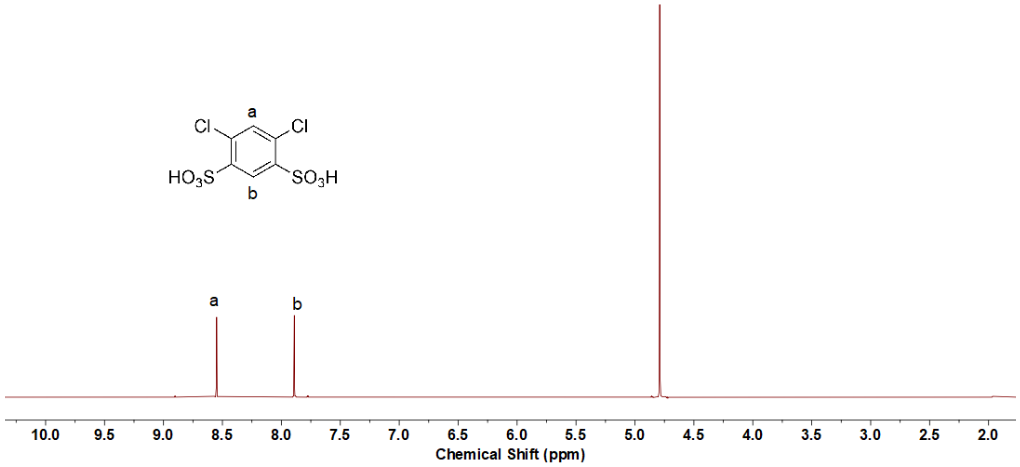

Fig. S 21 ^{1}H NMR in D_2O of pure 4,6-dichloro-1,3-benzenedisulfonic acid (7)

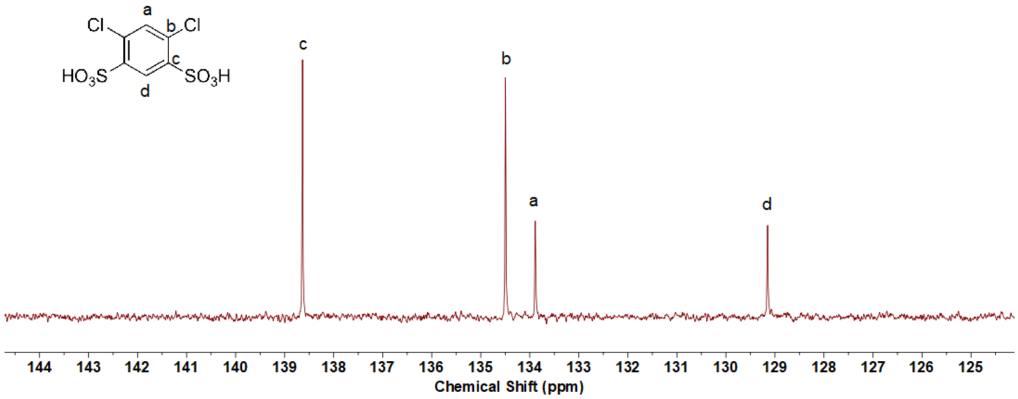

Fig. S 22 ^{13}C NMR in D_2O of pure 4,6-dichloro-1,3-benzenedisulfonic acid (7)

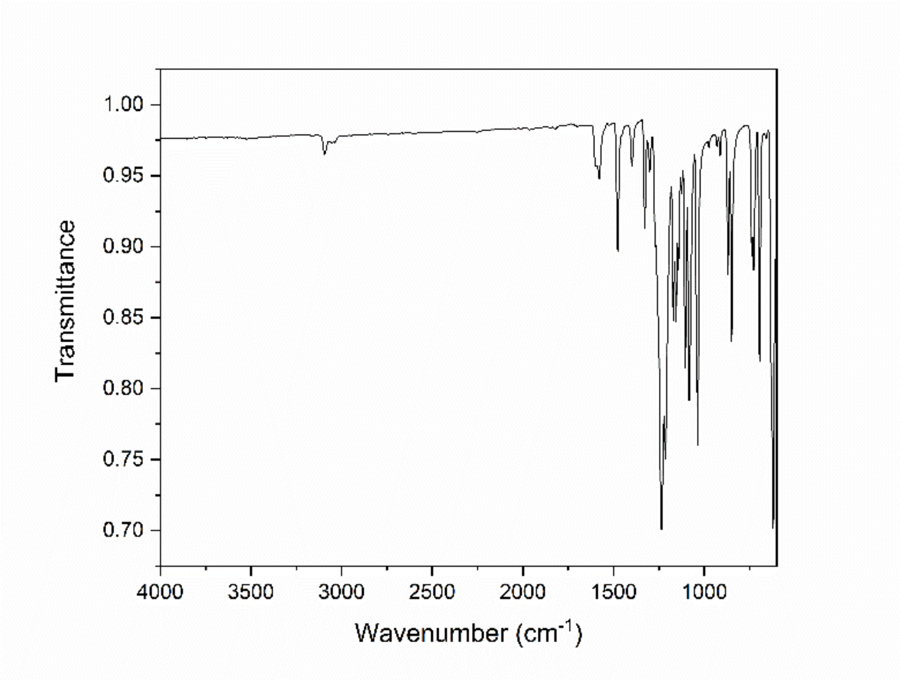

Fig. S 23 IR spectrum of sDFDPS (1)

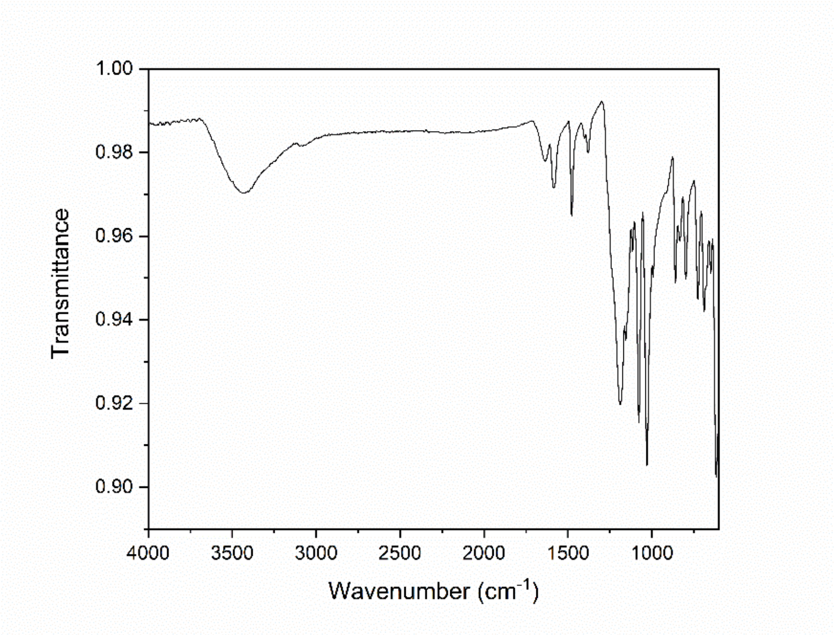

Fig. S 24 IR spectrum of sBFPPO (2)

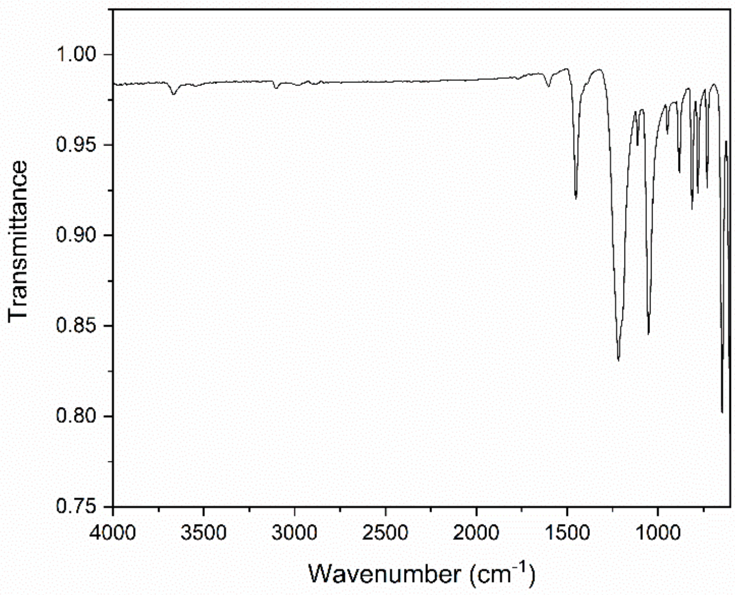

Fig. S 25 IR spectrum of 2,5-difluoro-1,3-benzenedisulfonic acid (3)

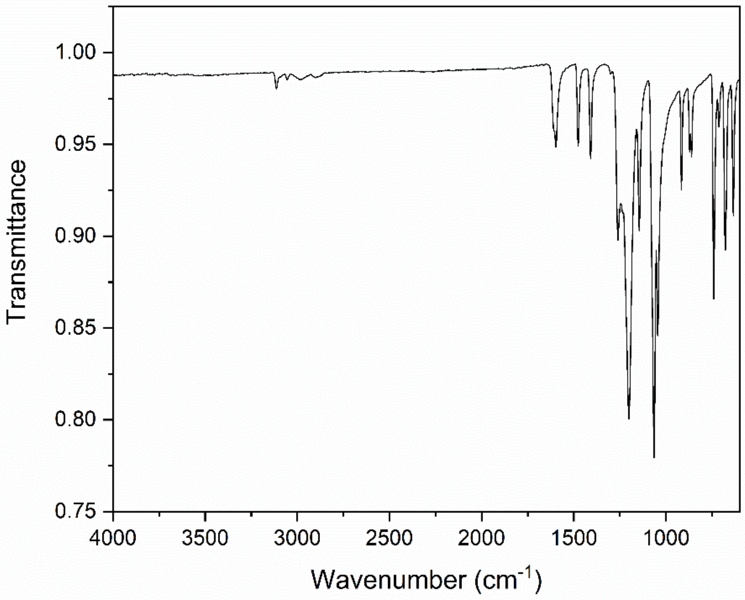

Fig. S 26 IR spectrum of 4,6-difluoro-1,3-benzenedisulfonic acid (4)

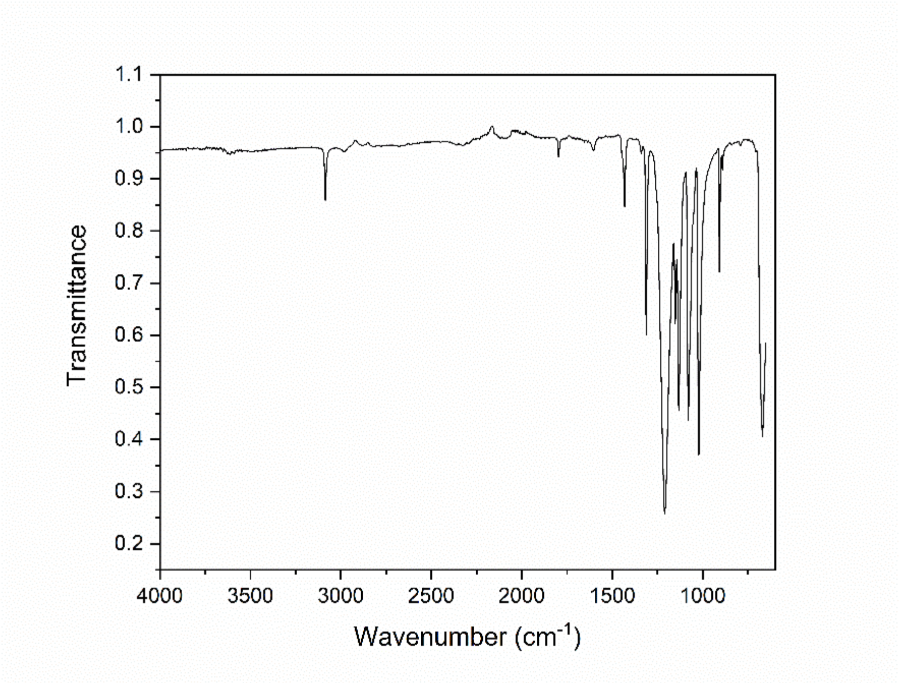

Fig. S 27 IR spectrum of 2,5-dibromo-1,4-benzenedisulfonic acid (5)

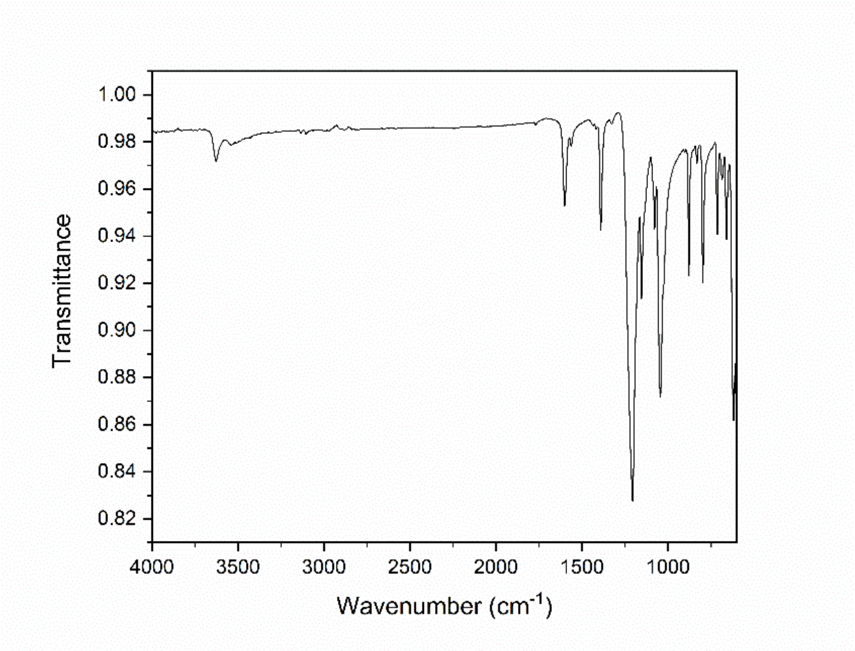

Fig. S 28 IR spectrum of the mixture of 2,5-dichlorobenzene-1,4-disulfonate (1,4-ds-2,5-DCB) and 2,5-dichlorobenzene-1,3-disulfonate (1,3-ds-2,5-DCB) (6)

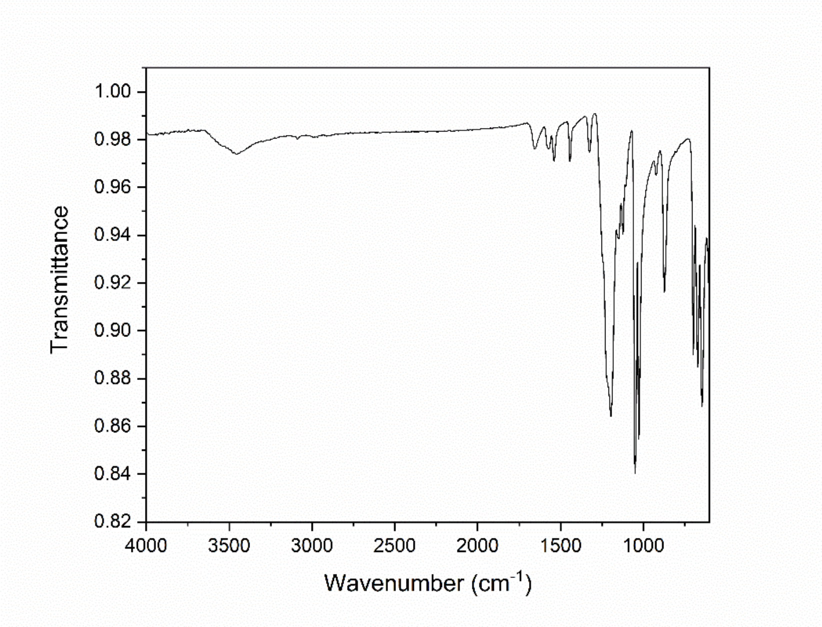

Fig. S 29 IR spectrum of 4,6-dichloro-1,3-benzenedisulfonic acid (7)

Sulfonation degree cross-verification by acid-base titration

Together with the ^1H NMR data, the degrees of sulfonation were cross-verified by acid-base titration following this general procedure: aqueous solutions of the sulfonated monomers were passed through an H-form ion-exchange resin twice. Water was removed using a rotary evaporator, followed by drying in a vacuum oven for 96 hours to achieve a constant weight. The dried sample, with a known weight, was then dissolved in water and titrated with 0.1 M NaOH. EW is calculated following the below given equation.

$$EW = \frac{1000 \cdot m}{V_{NaOH} \cdot C_{NaOH}}$$

Where, V_{NaOH} — volume of NaOH used for titration (mL), C_{NaOH} — molar concentration of NaOH ($mol \cdot L^{-1}$), m – mass of the dried sulfonated monomer in H-form (g)

The obtained equivalent weights closely matched the theoretically expected values (Table S1).

Table S 1. Theoretical and experimental equivalent weights of obtained monomers

**Monomer**	**$EW_{theor.}$**	**$EW_{Exp.}$**
sDFDPS (1)	207.18	207.04
sBFPPO (2)	184.81	184.16
ds-1,4-DFB (3)	137.11	136.79
ds-1,3-DFB (4)	137.11	-*
ds-1,4-DBB (5)	198.01	197.83
1,4-ds-2,5-DCB/1,3-ds-2,5-DCB (6)	153.56	153.22
ds-1,3-DCB (7)	153.56	-*

** Titrations for compounds **4** and **7** were omitted due to their high yields and the clarity of the ^1H NMR spectra.*
